# Multisensor Estimation Fusion on Statistical Manifold

**DOI:** 10.3390/e24121802

**Published:** 2022-12-09

**Authors:** Xiangbing Chen, Jie Zhou

**Affiliations:** 1 Division of Mathematics, Sichuan University Jinjiang College, Meishan 620860, China; 2 College of Mathematics, Sichuan University, Chengdu 610064, China

**Keywords:** distributed estimation fusion, elliptical distribution, information geometry, Manhattan distance, lie algebra

## Abstract

In the paper, we characterize local estimates from multiple distributed sensors as posterior probability densities, which are assumed to belong to a common parametric family. Adopting the information-geometric viewpoint, we consider such family as a Riemannian manifold endowed with the Fisher metric, and then formulate the fused density as an informative barycenter through minimizing the sum of its geodesic distances to all local posterior densities. Under the assumption of multivariate elliptical distribution (MED), two fusion methods are developed by using the minimal Manhattan distance instead of the geodesic distance on the manifold of MEDs, which both have the same mean estimation fusion, but different covariance estimation fusions. One obtains the fused covariance estimate by a robust fixed point iterative algorithm with theoretical convergence, and the other provides an explicit expression for the fused covariance estimate. At different heavy-tailed levels, the fusion results of two local estimates for a static target display that the two methods achieve a better approximate of the informative barycenter than some existing fusion methods. An application to distributed estimation fusion for dynamic systems with heavy-tailed process and observation noises is provided to demonstrate the performance of the two proposed fusion algorithms.

## 1. Introduction

Multisensor data fusion has been studied and widely applied to many important areas, such as image processing [1,2], wireless sensor networks [3,4] and remote sensing [5,6]. The research mainly centers on two basic architectures, i.e., centralized fusion and distributed fusion. The latter has less communication burden, higher flexibility and reliability, but challenges in dealing with the cross-correlation among local estimation errors caused by common process noises, related measurement noises and past data exchanges [7,8,9].

Considering the limitations of traditional Bayesian estimation methods such as some Kalman filtering variants, many estimation fusion methods only utilize the first two moments (i.e., mean and covariance) of each local posterior density for data fusion. For instance, the covariance intersection (CI) fusion method [10,11] propagates each local information pair consisting of the information matrix (i.e., the inverse covariance matrix) and the information vector (i.e., the state estimate left multiplied by the information matrix), and then provides a convex combination of all local information pair with specified normalized weights to yield a conservative fused estimate. Specifically, the determinant-minimization CI (DCI) method [12] selects weights by minimizing the trace or the determinant of fused covariance matrix, which also provided a set-theoretic interpretation [13]. Applying the set-theoretic criterion, some novel set-theoretic methods such as the relaxed Chebyshev center CI (RCC-CI) [14] and the analytic center CI (AC-CI) [15] take both the local state estimate and covariance matrix of estimation error into account to obtain the optimal weights, but the DCI only considers the latter.

The probability density function (PDF) constitutes a complete probabilistic description of the state estimate (i.e., the point estimate of the quantity of concern) from each sensor, which includes a lot of important information, such as support, tail decay and multimodality, so it is more suitable for fusion than the first two moments. We note that there are already many fusion methods which combine the local estimates together in the form of PDFs. As examples, under Gaussian assumptions, ref. [16] minimizes the Shannon entropy of a log-linear combination of all local PDFs, and provides the fused estimates with the same form as the DCI, but in general with different weights; the fast CI (FCI) algorithm [17] finds the “halfway” point between two local PDFs through minimizing Chernoff information, which has been generalized to the case of multiple sensors; The Kullback–Leibler averaging (KLA) algorithm [18] seeks an average PDF to minimize the sum of KL divergences between such average and local posterior densities.

Although many PDF fusion rules have been established and found to be useful in specific applications, there is no consistent theoretical basis for the principles of these rules and their possible alternatives. Moreover, ref. [19] illustrates that the space of PDFs is generally not Euclidean. For these reasons, information geometry regards the parametric space of PDFs as a statistical manifold with a Riemannian structure, and then studies the intrinsic properties by the tools of modern differential geometry. The theoretical framework can be exploited for the PDF fusion by assuming that all local posterior PDFs as points belong to a common Riemannian manifold. For example, ref. [20] formulates the fusion result as the Wasserstein barycenter by minimizing the sum of its squared Wasserstein distances to Gaussian inputs. The Wasserstein distance as a geodesic distance on the Riemannian manifold equipped with the Wasserstein metric is usually used for optimal transportation [21,22], while the Fisher metric applies primarily to information science. Therefore, by endowing such space with a natural Riemannian structure (i.e., the Fisher metric and Levi–Civita connection), the resulting geodesic distance, also called the Rao distance, has been taken as an intrinsic measure for the dissimilarity between two PDFs and then applied in wide fields such as neural networks [23,24], signal processing [25,26], and statistical inference [27,28].

In addition, due to the intrinsic mechanism of applications, uncertain modeling errors and the existence of outliers, the class of multivariate elliptical distributions (MEDs) including multivariate Gaussian distribution (MGD), multivariate generalized Gaussian distribution (MGGD) [29], multivariate *t*-distribution (MTD) [30], symmetric multivariate Laplace distribution [31], contaminated Gaussian distribution, Gaussian–Student’s *t* mixture distribution and so on, has enjoyed a wide range of applications [32,33,34,35,36,37,38,39,40,41]. To our knowledge, the application of information geometry in multisensor estimation fusion has not been studied in depth except two previous papers [42,43]. The geodesic projection (GP) method [42] for distributed estimation fusion, minimizing the sum of geodesic projection distances onto the Gaussian submanifold with a fixed mean vector, formulates the fused PDF as an informative barycenter of the Gaussian manifold. And the QMMHD fusion algorithm [43] extends the PDF fusion on the Gaussian manifold to the MED manifold by minimizing the sum of Manhattan distances (MHDs) between the fused density and each local posterior density on the Riemannian manifold of MEDs; however, it suffers from two major drawbacks. One is that the covariance estimate of the fused PDF is not specially designed, just using the same form as the CI estimate, and the other is that the convergence of the QMMHD algorithm can not be guaranteed in theory. At present, an in-depth research on the PDF fusion of non-Gaussian manifolds is quite lacking. Our goal is to develop efficient distributed estimation fusion algorithms for various scenarios and applications under a unified MED fusion framework, rather than considering a single fusion rule or method for a particular MED.

In the work, using the minimal MHD instead of the geodesic distance as the loss function of geometric fusion criterion, we propose two distributed fusion methods under the MED assumptions. The main contributions are summarized as follows:

(i) To exploit the non-Euclidean characteristics of probabilistic space and the decouple feature of Manhattan distance, we formulate a novel information-geometric criterion for fusion and discuss its inherent advantages.
(ii) We derive the explicit expression for the MHD-based projection from a point onto the MED submanifold with a fixed mean, and then develop two fusion methods MHDP-I and MHDP-E by relaxing the fusion criterion, which both have the same mean estimation fusion, but differ in the form of the fused covariance. A fixed point iteration is given to obtain this fused mean.
(iii) The MHDP-I obtains the fused covariance using a robust fixed point iterative algorithm with theoretical convergence, while the MHDP-E provides an explicit expression for the fused covariance by introducing a Lie algebraic structure. We also provide a crucial theorem presenting the exponential mapping and logarithmic mapping on the MED submanifold with a fixed mean.
(iv) Simulations indicate that the two proposed information-geometric methods outperform some existing fusion methods under non-Gaussian distributions.

The outline of this paper is organized as follows. In Section 2, the basic facts for information geometry and some useful results concerning the MED manifold are introduced. Section 3 formulates an information-geometric fusion criterion based on the minimal MHD, and then proposes the MHDP-I and MHDP-E fusion methods in Section 4. Numerical examples in Section 5 are provided to demonstrate the superiority of the two methods. Section 6 gives a conclusion. All proofs are given in the Appendix A, Appendix B, Appendix C, Appendix D, Appendix E, Appendix F.

### Notations

Throughout this paper, we use lightface letters to represent scalars and scalar-valued mappings, boldface lowercase letters to represent vectors, and boldface capital letters to represent matrices and matrix-valued mappings. All vectors are column vectors. The notation $S_{+}^{m}$ is the set of m×m real symmetric positive-definite matrices, $R_{+}$ is the space of all positive real numbers, and $R^{m}$ denotes the set of all *m*-dimensional real column vectors. The symbol $I_{m}$ stands for the identity matrix of order *m*. For a matrix A, $A^{T}$ and tr(A) denote its transpose and trace, respectively. Moreover, Exp(·), Log(·) and arccosh(·) are matrix exponential, matrix logarithm and inverse hyperbolic functions, respectively.

## 2. Preliminaries

In this section, we review basic notions in the fields of information geometry and present some important results for later use.

### 2.1. Statistical Manifold and Fisher Metric

Consider a statistical model

(1) $$\begin{array}{c} S=\left\{p\left(x;\theta \right):\theta =\left(\theta^{1},\ldots ,\theta^{m}\right)\in \Theta \right\} \end{array}$$

of probability densities with the global coordinate system $\theta =(\theta^{1},\ldots ,\theta^{m})$, where Θ is an open set of $R^{m}$. Using the Fisher metric *g* induced by the m×m Fisher information matrix with the (i,j)-th entry

(2) $$\begin{array}{c} g_{ij}\left(\theta \right)=E_{\theta }\left[\frac{\partial }{\partial \theta^{i}}\log p\left(x;\theta \right)\frac{\partial }{\partial \theta^{j}}\log p\left(x;\theta \right)\right], \end{array}$$

where $E_{\theta }\left[\cdot \right]$ represents the expectation operator with respect to p(x;θ), the statistical manifold S can be endowed with a natural Riemannian differentiable structure [44]. For brevity, we shall abbreviate the point p(x;θ) of S as its coordinate θ.

Let $T_{\theta }S$ be the tangent vector space at the point θ in S, and an inner product on $T_{\theta }S$ is then defined by the Fisher metric *g*, written as

(3) $$\begin{array}{c} {\langle \cdot ,\cdot \rangle }_{\theta }:T_{\theta }S\times T_{\theta }S\to R. \end{array}$$

Thus, the geodesic distance between two points $\theta_{0}$ and $\theta_{1}$ in S, also called the Rao distance or Fisher information distance, can be obtained through solving the following functional minimization problem

(4) $$\begin{array}{c} \ell_{F}\left(\theta_{0},\theta_{1}\right):=\min_{\theta \in \Gamma }\int_{0}^{1}\sqrt{{\langle \dot{\theta }\left(t\right),\dot{\theta }\left(t\right)\rangle }_{\theta (t)}}dt, \end{array}$$

where the dot symbol over a variable signifies its derivative with respect to t∈R, and the set Γ consists of all piecewise smooth curves linking $\theta_{0}$ to $\theta_{1}$.

For any $\nu \in T_{\theta }S$, there exists a unique geodesic segment $\gamma \left(t;\theta_{0},\nu \right),t\in \left[0,1\right]$, satisfying $\gamma \left(0\right)=\theta_{0}$ and $\dot{\gamma }\left(0\right)=\nu$, and then the exponential mapping is defined as

(5) $$\begin{array}{cc} \exp_{\theta_{0}}:T_{\theta_{0}}S & \to S\\ \nu & \mapsto \exp_{\theta_{0}}\nu =\gamma \left(1;\theta_{0},\nu \right). \end{array}$$

Conversely, the logarithmic mapping $\log_{\theta_{0}}\left(\theta_{1}\right)$ maps $\theta_{1}$ into the tangent vector ν at $\theta_{0}$. As a generalization, the manifold retraction R(·) is a smooth mapping from the tangent bundle $TS=\{T_{\theta }S:\theta \in \Theta \}$ into S (see, e.g., [45]), and its restriction $R_{\theta }\left(\cdot \right)$ on an open ball B(0,r) with radius *r* in $T_{\theta }S$ satisfies $R_{\theta }\left(0\right)=\theta$ and $dR_{\theta }{|}_{0}={\mathrm{id}}_{T_{\theta }S}$. Moreover, its inverse mapping $R_{\theta }^{-1}\left(\cdot \right)$, called the lifting mapping, exists in a neighborhood of θ. In particular, the exponential mapping and logarithmic mapping are the commonly used retraction mapping and lifting mapping, respectively.

### 2.2. Information Geometry of Elliptical Distributions

An *m*-dimensional random vector x has multivariate elliptical distribution ${\mathrm{EL}}_{h}^{m}\left(\mu ,\Sigma \right)$, if its probability density function is of the form

(6) $$p_{h}\left(x;\mu ,\Sigma \right)={\left|\Sigma \right|}^{-1/2}h\left({\left(x-\mu \right)}^{T}\Sigma^{-1}\left(x-\mu \right)\right)$$

with some generating function h(·), where $\mu \in R^{m}$ is mean vector and $\Sigma \in S_{+}^{m}$ is positive-definite scatter matrix. From [46], x has covariance matrix $\kappa_{h}\Sigma$ with scale parameter $\kappa_{h}$. As two examples, the generating functions of the MGGD with shape parameter β and the MTD with ν degrees of freedom, respectively, are

(7) $$\begin{array}{c} h\left(u\right)=\frac{\beta \Gamma (m/2)}{\pi^{m/2}\Gamma \left(m/\left(2\beta \right)\right)2^{m/(2\beta )}}\exp\left(-\frac{1}{2}u^{\beta }\right) \end{array}$$

and

(8) $$\begin{array}{c} h\left(u\right)=\frac{\Gamma ((\nu +m)/2)}{{\left(\pi \nu \right)}^{m/2}\Gamma \left(\nu /2\right)}{\left(1+\frac{u}{\nu }\right)}^{-(\nu +m)/2}. \end{array}$$

Suppose that Ω is the lower-triangle Cholesky factor of Σ, $z=\Omega^{-1}\left(x-\mu \right)$ and $\xi ={d\log(h(∥z∥}^{2}))/{d(∥z∥}^{2})$. Denote

(9) $$\begin{array}{c} a_{h}=\frac{1}{m}E[\xi^{2}{∥z∥}^{2}],\;b_{h}=\frac{1}{m(m+2)}E[\xi^{2}{∥z∥}^{4}]. \end{array}$$

It is easy to deduce that

(i) for the MGGD, $a_{h}=\beta^{2}\Gamma \left(2+m/(2\beta )-1/\beta \right)/$$\left(2^{1/\beta }m\Gamma \left(m/\left(2\beta \right)\right)\right)$, $b_{h}=\left(m+2\beta \right)/\left(4m+8\right)$;
(ii) and for the MTD, $a_{h}=b_{h}=\left(\nu +m\right)/\left(4\left(\nu +m+2\right)\right)$.

**Remark** **1.** *For other classical elliptical distributions, such as the Pearson-type VII class of distributions, the multivariate Cauchy distribution and a special subfamily of MEDs, the reference [47] has derived the analytic forms for $a_{h}$ and $b_{h}$. However, in practical applications, some special MEDs (e.g., the Gaussian mixture distribution and the Gaussian–Student’s t mixture distribution [41]) used to model the non-Gaussian distributions may not have their analytical forms, so we can use (9) to numerically calculate the two parameters, or approximate these MEDs using some specified classical elliptical distributions.*

By introducing a Riemannian structure associated with the Fisher metric, the MED manifold

(10) $$\begin{array}{c} M=\left\{{\mathrm{EL}}_{h}^{m}\left(\mu ,\Sigma \right):\mu \in R^{m},\Sigma \in S_{+}^{m}\right\} \end{array}$$

with the same fixed function *h* can be regarded as a Riemannian manifold with coordinate system (μ,Σ). Note that the dimension of M is m+m(m+1)/2. From [47], the squared line element at a point θ=(μ,Σ) in M is

(11) $$\begin{array}{c} ds^{2}=4a_{h}d\mu^{T}\Sigma^{-1}d\mu +2b_{h}\mathrm{tr}{\left(\Sigma^{-1}d\Sigma \right)}^{2}+\frac{4b_{h}-1}{4}{\mathrm{tr}}^{2}\left(\Sigma^{-1}d\Sigma \right), \end{array}$$

and the geodesic equations are given by

(12) $$\begin{array}{cc} \; & \ddot{\mu }-\dot{\Sigma }\Sigma^{-1}\dot{\mu }=0, \end{array}$$

(13) $$\begin{array}{cc} \; & \ddot{\Sigma }+a\dot{\mu }{\dot{\mu }}^{T}-b{\dot{\mu }}^{T}\Sigma^{-1}\dot{\mu }\Sigma -\dot{\Sigma }\Sigma^{-1}\dot{\Sigma }=0, \end{array}$$

where $a=a_{h}/b_{h}$ and $b=a_{h}\left(4b_{h}-1\right)/\left(\left(8+4m\right)b_{h}^{2}-mb_{h}\right)$.

Next, we derive the explicit expressions of the geodesic distance and geodesic curve between two given endpoints on the two-dimensional MED manifold M (i.e., m=1) with coordinate system $\left(\mu ,\sigma^{2}\right)$, which will be used in Section 5.1.

**Theorem** **1.** *Consider the two-dimensional MED manifold M and two points $p_{1}={\mathrm{EL}}_{h}^{1}\left(\mu_{1},\sigma_{1}^{2}\right)$ and $p_{2}={\mathrm{EL}}_{h}^{1}\left(\mu_{2},\sigma_{2}^{2}\right)$ in M. Let $c_{h}=8a_{h}/\left(12b_{h}-1\right)$ and $d_{h}=\sqrt{12b_{h}-1}$, then the geodesic distance between $p_{1}$ and $p_{2}$ is*

(14) $$\begin{array}{c} \ell_{F}=d_{h}\cdot \mathrm{arccosh}\left(\frac{c_{h}{\left(\mu_{2}-\mu_{1}\right)}^{2}+2\left(\sigma_{1}^{2}+\sigma_{2}^{2}\right)}{4\sigma_{1}\sigma_{2}}\right). \end{array}$$

*Additionally, the geodesic curve between $p_{1}$ and $p_{2}$ is given by*

*(i)*  *If $\mu_{1}=\mu_{2}$, then*
(15) $$\begin{array}{cc} \mu (s) & =\mu_{1}=\mu_{2}, \end{array}$$
(16) $$\begin{array}{cc} \sigma^{2}\left(s\right) & =\sigma_{1}^{2}\cdot \exp\left(2s/d_{h}\right); \end{array}$$
*(ii)*  *If $\mu_{1}\neq \mu_{2}$, then*
(17) $$\begin{array}{cc} \mu (s) & =\frac{1}{\sqrt{c_{h}}}\left(\delta_{1}+2\delta_{2}\tanh\left(s/d_{h}+\varepsilon \right)\right), \end{array}$$
(18) $$\begin{array}{cc} \sigma^{2}\left(s\right) & =2\delta_{2}^{2}\cosh^{-2}\left(s/d_{h}+\varepsilon \right), \end{array}$$
  *where,*
(19) $$\begin{array}{cc} \delta_{1} & =\frac{2\sigma_{2}^{2}+c_{h}\mu_{2}^{2}-2\sigma_{1}^{2}-c_{h}\mu_{1}^{2}}{2\sqrt{c_{h}}\left(\mu_{2}-\mu_{1}\right)}, \end{array}$$
(20) $$\begin{array}{cc} \delta_{2} & =\frac{1}{2}\sqrt{2\sigma_{1}^{2}+{\left(\sqrt{c_{h}}\mu_{1}-\delta_{1}\right)}^{2}}, \end{array}$$
(21) $$\begin{array}{cc} \varepsilon & =\mathrm{arctanh}\left(\frac{\sqrt{c_{h}}\mu_{1}-\delta_{1}}{2\delta_{2}}\right). \end{array}$$

**Proof.**  See Appendix A.    □

However, in the general case for m>1, how to obtain the closed form of Rao distance on the MED manifold M remains an unsolved problem, except for some submanifolds with a constant mean vector or constant scatter matrix (see, e.g., [48]).

### 2.3. Some Submanifolds of Elliptical Distributions

One of the commonly used submanifolds of M is

(22) $$\begin{array}{c} M_{\left[\mu_{0},\cdot \right]}=\left\{{\mathrm{EL}}_{h}^{m}\left(\mu_{0},\Sigma \right):\Sigma \in S_{+}^{m}\right\} \end{array}$$

having a fixed mean $\mu_{0}$ and the coordinate system $\left(\mu_{0},\Sigma \right)$ or Σ for short. It is a totally geodesic submanifold, meaning that each geodesic of $M_{\left[\mu_{0},\cdot \right]}$ is also a geodesic of M. As shown in [48], for any two tangent vectors $\nu_{1},\nu_{2}\in T_{\theta }\left(M_{\left[\mu_{0},\cdot \right]}\right)$ at a point $\theta =(\mu_{0},\Sigma )$, the Fisher inner product is given by

(23) $$\begin{array}{c} {\langle \nu_{1},\nu_{2}\rangle }_{\theta }=2b_{h}\mathrm{tr}\left(\Sigma^{-1}\nu_{1}\Sigma^{-1}\nu_{2}\right)+\frac{4b_{h}-1}{4}\mathrm{tr}\left(\Sigma^{-1}\nu_{1}\right)\mathrm{tr}\left(\Sigma^{-1}\nu_{2}\right), \end{array}$$

and the Rao distance between two points $\theta_{0}=\left(\mu_{0},\Sigma_{0}\right)$ and $\theta_{1}=\left(\mu_{0},\Sigma_{1}\right)$ in $M_{\left[\mu_{0},\cdot \right]}$ equals

(24) $$\begin{array}{c} \ell_{\Sigma }\left(\theta_{0},\theta_{1}\right)=\sqrt{2b_{h}\mathrm{tr}\left({\mathrm{Log}}^{2}\left(\Sigma_{0}^{-1}\Sigma_{1}\right)\right)+\frac{4b_{h}-1}{4}{\mathrm{tr}}^{2}\left(\mathrm{Log}\left(\Sigma_{0}^{-1}\Sigma_{1}\right)\right)}. \end{array}$$

Another special submanifold is defined as

(25) $$\begin{array}{c} M_{\left[\cdot ,\alpha \Sigma_{0}\right]}=\left\{{\mathrm{EL}}_{h}^{m}\left(\mu ,\alpha \Sigma_{0}\right):\mu \in R^{m},\alpha \in R_{+}\right\} \end{array}$$

with a fixed scatter matrix $\Sigma_{0}\in S_{+}^{m}$ and the coordinate system $\left(\mu ,\alpha \Sigma_{0}\right)$ or (μ,α) for short. As [43], an explicit expression for the Rao distance on $M_{\left[\cdot ,\alpha \Sigma_{0}\right]}$ is given as follows.

**Lemma** **1.** *Given two points $\theta_{0}=\left(\mu_{0},\Sigma_{0}\right)$ and $\theta_{\alpha }=\left(\mu ,\alpha \Sigma_{0}\right)$ in $M_{\left[\cdot ,\alpha \Sigma_{0}\right]}$, let $c=8a_{h}/\left(4m\left(m+2\right)b_{h}-m^{2}\right)$ and $d_{M}={\left(\mu_{0}-\mu \right)}^{T}\Sigma_{0}^{-1}\left(\mu_{0}-\mu \right)$, then on $M_{\left[\cdot ,\alpha \Sigma_{0}\right]}$, the Rao distance between $\theta_{0}$ and $\theta_{\alpha }$ is*

(26) $$\begin{array}{c} \ell_{\mu }\left(\theta_{0},\theta_{\alpha }\right)=\sqrt{\frac{2a_{h}}{c}}\mathrm{arccosh}\left(\frac{{\left(cd_{M}+2\right)}^{2}}{8\alpha }+\frac{\alpha }{2}+\frac{cd_{M}}{2}\right). \end{array}$$

### 2.4. Manhattan Distances

At present, there is no explicit expression for the Rao distance on the MED manifold M. Intuitively motivated from the commonly adopted distance to measure the length of path along the coordinate curves on M as the $\ell_{1}$ distance in Euclidean space, we introduce an intermediate point $\theta_{\alpha }=\left(\mu_{1},\alpha \Sigma_{0}\right)\in M$ to link $\theta_{0}=\left(\mu_{0},\Sigma_{0}\right)$ and $\theta_{1}=\left(\mu_{1},\Sigma_{1}\right)$ for $\alpha \in R^{+}$, and then construct a one-parametric class $A=\{\ell_{\alpha }\left(\theta_{0},\theta_{1}\right):\alpha \in R_{+}\}$ of MHDs from two Rao distances, where each member in A satisfies

(27) $$\begin{array}{c} \ell_{\alpha }\left(\theta_{0},\theta_{1}\right):=\ell_{\mu }\left(\theta_{0},\theta_{\alpha }\right)+\ell_{\Sigma }\left(\theta_{\alpha },\theta_{1}\right)\geq \ell_{F}\left(\theta_{0},\theta_{1}\right). \end{array}$$

Moreover, for providing tighter upper bounds for the Rao distance $\ell_{F}\left(\theta_{0},\theta_{1}\right)$ on M, the minimal MHD

(28) $$\begin{array}{c} \ell_{\hat{\alpha }}\left(\theta_{0},\theta_{1}\right)=\min_{\alpha \in R_{+}}\ell_{\alpha }\left(\theta_{0},\theta_{1}\right) \end{array}$$

as the minimum in the class A can be obtained by optimally seeking the parameter $\hat{\alpha }$.

## 3. Fusion Criterion

Consider a distributed system with *n* sensors observing a common state $x\in R^{m}$. As many practical scenarios for dynamic target tracking in [49,50], the dynamic system is assumed having the heavy-tailed process and observation noises. We then adopt the MED assumption to better exploit heavy-tailed features inherent in noises, and denote by $p_{k}={\mathrm{EL}}_{h}^{m}\left({\hat{x}}_{k},P_{k}\right)$ the local posterior density from the *k*-th sensor. The goal is to fuse all $p_{k}$ into a single fused density with unavailable correlations among local sensors.

### 3.1. Information-Geometric Criterion

In practice, the Kullback–Leibler divergence (KLD) and Rao distance are widely accepted as basic information theoretic quantities that can capture higher-order statistical information, but the former is not a true distance in mathematics owing to the lack of triangle inequality and symmetry. Instead, as a natural intrinsic measure, the Rao distance not only enables us to deal with statistical issues while respecting the nonlinear geometry structure of MEDs, but is also invariant under coordinate transformations. From the viewpoint of information geometry, the geometrical illustration develops an intrinsic understanding of statistical models, and also provides a better avenue for estimation fusion. Then, taking the sum of geodesic distances $\ell_{F}\left(p_{k},p\right)$ between $p={\mathrm{EL}}_{h}^{m}\left(\mu ,\Sigma \right)$ and all $p_{k}$ in the MED manifold M as the cost function, we formulate a fusion criterion as

(29) $$\begin{array}{c} \left(\hat{x},P\right)=\underset{\mu \in R^{m},\Sigma \in S_{+}^{m}}{\arg\min}\sum_{k=1}^{n}\ell_{F}\left(p_{k},p\right) \end{array}$$

to fuse all local available information $p_{k}$ into a single posterior density $\hat{p}={\mathrm{EL}}_{h}^{m}\left(\hat{x},P\right)$.

The aforementioned inherent feature of the Rao distance ensures a unique fusion result in the multisensor fusion network involving different measurement frameworks. However, due to the absence of the closed form of Rao distance on the manifold of MEDs, the minimal MHD (28) as its tight upper bound is applied in the fusion criterion (29), that is,

(30) $$\begin{array}{cc} \left(\hat{x},P\right) & =\underset{\mu \in R^{m},\Sigma \in S_{+}^{m}}{\arg\min}\sum_{k=1}^{n}\min_{\alpha_{k}\in R_{+}}\ell_{\alpha_{k}}\left(p_{k},p\right)\\ \; & =\underset{\mu \in R^{m},\Sigma \in S_{+}^{m}}{\arg\min}\sum_{k=1}^{n}\min_{\alpha_{k}\in R_{+}}\left(\ell_{\mu }\left(p_{k},p_{\alpha_{k}}\right)+\ell_{\Sigma }\left(p_{\alpha_{k}},p\right)\right), \end{array}$$

where the intermediate points $p_{\alpha_{k}}={\mathrm{EL}}_{h}^{m}\left(\mu ,\alpha_{k}P_{k}\right)$.

The advantages of determining the minimal MHD to measure the dissimilarity between two MEDs are obvious:

(i) The minimal MHD (28) is constructed from two Rao distances on two submanifolds (22) and (25) by inserting an intermediate point, thus having the decouple feature and inheriting some good properties from the Rao distance.
(ii) As a tight upper bound for the Rao distance on the MED manifold, the minimal MHD (28) can be efficiently computed numerically. Moreover, its superior approximation performance has been fully verified by comparison with the Rao distances on the manifolds of MTDs and MGGDs (see [43] for more details).

### 3.2. Decoupling Fusion Criterion

Define the MHD-based projection distance (MHDPD) from a point $p_{0}={\mathrm{EL}}_{h}^{m}\left({\hat{x}}_{0},P_{0}\right)$ in M onto the submanifold $M_{\left[\mu ,\cdot \right]}$ along the geodesic in $M_{\left[\cdot ,\alpha P_{0}\right]}$ as

(31) $$\begin{array}{c} \ell_{M}\left(p_{0},M_{\left[\mu ,\cdot \right]}\right):=\min_{\alpha \in R_{+}}\ell_{\mu }\left(p_{0},p_{\alpha }\right), \end{array}$$

where $p_{\alpha }={\mathrm{EL}}_{h}^{m}\left(\mu ,\alpha P_{0}\right)$.

Before further solving the optimization problem (30), we firstly derive the explicit expressions of the MHD-based projection (MHDP) from a point onto the submanifold $M_{\left[\mu ,\cdot \right]}$ and the corresponding MHDPD.

**Theorem** **2.** *The MHDPD from $p_{0}={\mathrm{EL}}_{h}^{m}\left({\hat{x}}_{0},P_{0}\right)$ in M onto the submanifold $M_{\left[\mu ,\cdot \right]}$ is given as*

(32) $$\begin{array}{c} \ell_{M}\left(p_{0},M_{\left[\mu ,\cdot \right]}\right)=\sqrt{\frac{2a_{h}}{c}}\mathrm{arccosh}\left(cd_{0}+1\right) \end{array}$$

*with $d_{0}={\left({\hat{x}}_{0}-\mu \right)}^{T}P_{0}^{-1}\left({\hat{x}}_{0}-\mu \right)$, and the corresponding MHDP is $p_{{\hat{\alpha }}_{0}}={\mathrm{EL}}_{h}^{m}\left(\mu ,\Sigma \right)$ with the scatter matrix*

(33) $$\begin{array}{c} \Sigma ={\hat{\alpha }}_{0}P_{0} \end{array}$$

*and the optimal scale factor*

(34) $$\begin{array}{c} {\hat{\alpha }}_{0}=\frac{c}{2}d_{0}+1. \end{array}$$

**Proof.**  See Appendix B.    □

As an application to distributed dynamic system, each available local estimate $\left({\hat{x}}_{i},P_{i}\right)\in R^{m}\times S_{+}^{m}$ should be limited to a bounded local feasible set $S_{i}$ around the true location $\left(\mu_{i},\Sigma_{i}\right)$ with high reliability (see, e.g., [51]). Therefore by the property of continuous function on compact set, it is straightforward to decompose the optimization (30) into two steps–first over μ and then over Σ:

(35) $$\begin{array}{c} \min_{\mu \in R^{m}}\min_{\Sigma \in S_{+}^{m}}\sum_{k=1}^{n}\min_{\alpha_{k}\in R_{+}}\left(\ell_{\mu }\left(p_{k},p_{\alpha_{k}}\right)+\ell_{\Sigma }\left(p_{\alpha_{k}},p\right)\right). \end{array}$$

Denote two functions of the variable $\mu \in R^{m}$ as follows:

(36) $$\begin{array}{cc} d_{k}\left(\mu \right) & ={\left({\hat{x}}_{k}-\mu \right)}^{T}P_{k}^{-1}\left({\hat{x}}_{k}-\mu \right), \end{array}$$

(37) $$\begin{array}{cc} {\hat{\alpha }}_{k}\left(\mu \right) & =\frac{c}{2}d_{k}\left(\mu \right)+1. \end{array}$$

Similar to the relaxing strategy in [42], we solve the optimization (35) by successively optimizing Σ and μ. Specifically, exchanging the summation and the minimization over Σ and simplifying the objective function in the summation, (35) is then reduced to

(38) $$\begin{array}{c} \hat{x}=\underset{\mu \in R^{m}}{\arg\min}\sum_{k=1}^{n}\min_{\alpha_{k}\in R_{+}}\ell_{\mu }\left(p_{k},p_{\alpha_{k}}\right), \end{array}$$

or equivalently from (31), (32) and (34),

(39) $$\begin{array}{cc} \hat{x} & =\underset{\mu \in R^{m}}{\arg\min}\sum_{k=1}^{n}\ell_{M}\left(p_{k},M_{\left[\mu ,\cdot \right]}\right)\\ \; & =\underset{\mu \in R^{m}}{\arg\min}\sum_{k=1}^{n}\mathrm{arccosh}\left(cd_{k}\left(\mu \right)+1\right), \end{array}$$

where $\hat{\alpha }=\left({\hat{\alpha }}_{1},\ldots ,{\hat{\alpha }}_{n}\right)$ with

(40) $$\begin{array}{c} {\hat{\alpha }}_{k}={\hat{\alpha }}_{k}\left(\hat{x}\right)=\frac{c}{2}d_{k}\left(\hat{x}\right)+1,\;k=1,\ldots ,n. \end{array}$$

**Figure 1:**
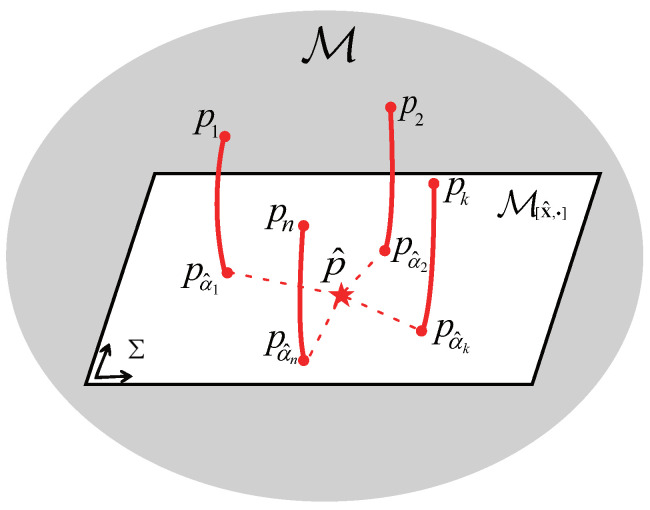
The fusion of *n* local posterior densities based on the MHDPs.

In general, (39) is not equivalent to the original optimization problem (35) owing to its providing a lower bound for the minimum of the objective function in (35), but it makes good sense for the mean fusion based on the following insights:

(i) As depicted in Figure 1, we replace the local posterior densities $p_{k}$ with the MHDPs $p_{{\hat{\alpha }}_{k}}={\mathrm{EL}}_{h}^{m}\left(\mu ,{\hat{\alpha }}_{k}P_{k}\right)$ on $M_{\left[\mu ,\cdot \right]}$ to measure the dissimilarity between the sought-for fused posterior density $\hat{p}$ and $p_{k}$ in the fusion criterion (30).
(ii) The inner minimizations in (39) project each local density $p_{k}$ onto the MED submanifold $M_{\left[\mu ,\cdot \right]}$ with some common fixed mean to obtain its substitute $p_{{\hat{\alpha }}_{k}}$. An appropriate candidate $\hat{x}$ for the specified mean variable μ is selected by the outer optimization, minimizing the sum of MHDPDs from the local posterior densities $p_{k}$ onto $M_{\left[\mu ,\cdot \right]}$.

Furthermore, the mean estimation fusion (39) obtains the final MHDPs $p_{{\hat{\alpha }}_{k}}={\mathrm{EL}}_{h}^{m}\left(\hat{x},{\hat{\alpha }}_{k}P_{k}\right)$ in $M_{\left[\hat{x},\cdot \right]}$ with the proper scale factors ${\hat{\alpha }}_{k}$. Additionally, moving each local estimate $\left({\hat{x}}_{k},P_{k}\right)$ to its MHDP $\left(\hat{x},{\hat{\alpha }}_{k}P_{k}\right)$ inevitably leads to an increase in the local estimation error. It is observed from (40) that ${\hat{\alpha }}_{k}\left(\hat{x}\right)$ will tend to the scalar 1 from the right hand side as the fused mean $\hat{x}$ approaches the *k*-th local mean estimate ${\hat{x}}_{k}$, so it is reasonable to replace the local scatter matrix $P_{k}$ with ${\hat{\alpha }}_{k}P_{k}$. Then, we can fuse all MHDPs $p_{{\hat{\alpha }}_{1}},\ldots ,p_{{\hat{\alpha }}_{n}}$ on the totally geodesic submanifold $M_{\left[\hat{x},\cdot \right]}$ with the fixed $\hat{x}$ to seek the fused scatter matrix P, using the following fusion criterion:

(41) $$\begin{array}{c} P=\underset{\Sigma \in S_{+}^{m}}{\arg\min}\sum_{k=1}^{n}\ell_{\Sigma }^{2}\left(p_{{\hat{\alpha }}_{k}},p\right). \end{array}$$

It is worth noting that the squared geodesic distance, used as the cost function in (41), differs from the original fusion criterion (29). The main reason are as follows:

(i) The covariance fusion (41) is indeed performed on the space $S_{+}^{m}$, since the Rao distance $\ell_{\Sigma }$ of $M_{\left[\hat{x},\cdot \right]}$ only depends on the coordinate $\Sigma \in S_{+}^{m}$. Moreover, the Riemannian mean (also called Riemannian center of mass) of data points $p_{1},\ldots ,p_{n}$ in $S_{+}^{m}$, which is the unique global minimizer of $\sum_{k=1}^{n}\ell^{2}\left(p_{k},p\right)$ with a geodesic distance ℓ(·,·) on $S_{+}^{m}$ (see [52,53]), has been widely studied on the manifold of covariance matrices [54].
(ii) The criterion $\sum_{k=1}^{n}\ell \left(p_{k},p\right)$ gives a robust alternative for the center of mass, called the Riemannian median [55,56]. Both the Riemannian mean and the Riemannian median have been successfully applied to signal detection (e.g., [57,58]) and have shown their own advantages. However when only two sensors (i.e., n=2) are considered, any point lying on the shortest geodesic segment between two points $p_{1}$ and $p_{2}$ can be regarded as the Riemannian median, which seems quite undesirable for fusion. In addition, the final mean fusion (39) does not contradict this claim owing to its adopting the MHDPD $\ell_{M}$ as the cost function.
(iii) Due to the intractable root operation within the geodesic distance $\ell_{\Sigma }$, the squared geodesic distance is considered for the convenience of solving the optimization problem (41). As illustrated in Section 4.2, we develop an efficient iterative strategy for the fused scatter estimate in the criterion (41) with at least linear convergence.

In summary, the final fused mean estimate $\hat{x}$ and scatter matrix estimate P can be obtained by solving (39) and (41), respectively.

## 4. Two MHDP-Based Fusion Methods

In this section, we first derive a fixed point iteration for the mean fusion (39), and then provide two different forms of covariance estimation fusion by iteratively solving the optimization problem (41) and introducing the Lie algebraic structure on $M_{\left[\hat{x},\cdot \right]}$ to obtain an explicit form of the fused covariance, respectively.

### 4.1. Mean Estimation Fusion

Define two auxiliary functions

(42) $$\begin{array}{cc} \phi (x) & =\mathrm{arccosh}(x),\;x\geq 1, \end{array}$$

(43) $$\begin{array}{cc} {\overset{˘}{d}}_{k}\left(x\right) & =cd_{k}\left(x\right)+1,\;x\in R^{m}, \end{array}$$

and then rewrite (39) as

(44) $$\begin{array}{c} \hat{x}=\underset{\mu \in R^{m}}{\arg\min}\sum_{k=1}^{n}\phi \left({\overset{˘}{d}}_{k}\left(\mu \right)\right). \end{array}$$

**Theorem** **3.** *The solution of the optimization problem (44) can be expressed in the following implicit form*

(45) $$\begin{array}{c} \hat{x}={\left(\sum_{k=1}^{n}\omega_{k}\left(\hat{x}\right)P_{k}^{-1}\right)}^{-1}\sum_{k=1}^{n}\omega_{k}\left(\hat{x}\right)P_{k}^{-1}{\hat{x}}_{k}, \end{array}$$

*where the normalized weights are given as*

(46) $$\begin{array}{c} \omega_{k}\left(\hat{x}\right)=\frac{\dot{\phi }\left({\overset{˘}{d}}_{k}\left(\hat{x}\right)\right)}{\sum_{k=1}^{n}\dot{\phi }\left({\overset{˘}{d}}_{k}\left(\hat{x}\right)\right)},\;k=1,\ldots ,n. \end{array}$$

**Proof.**  See Appendix C.    □

In Theorem 3, the fused mean estimate $\hat{x}$ given by (45) has the same form as the traditional CI estimate, but in general with different weights. To solve the implicit Equation (45) for $\hat{x}$, we derive a fixed point iteration for seeking the final $\hat{x}$ in the following theorem.

**Theorem** **4.** *By adopting the fixed point iteration*

(47) $$\begin{array}{c} \mu_{t+1}={\left(\sum_{k=1}^{n}{\hat{\omega }}_{k}\left(\mu_{t}\right)P_{k}^{-1}\right)}^{-1}\sum_{k=1}^{n}{\hat{\omega }}_{k}\left(\mu_{t}\right)P_{k}^{-1}{\hat{x}}_{k} \end{array}$$

*with the normalized weights*

(48) $$\begin{array}{c} {\hat{\omega }}_{k}\left(\mu_{t}\right)=\frac{{\left({\overset{˘}{d}}_{k}^{2}\left(\mu_{t}\right)-1\right)}^{-\frac{1}{2}}}{\sum_{k=1}^{n}{\left({\overset{˘}{d}}_{k}^{2}\left(\mu_{t}\right)-1\right)}^{-\frac{1}{2}}},\;k=1,\ldots ,n, \end{array}$$

*the resulting sequence $\left\{\mu_{t},\;t\in N\right\}$ converges to a solution $\hat{x}$ of (45) as t tends to infinity.*

**Proof.**  See Appendix D.    □

As shown in Algorithm 1, the fixed point iteration (47) is adopted to calculate the optimal estimate of mean $\hat{x}$ in (44), where the convergence of iteration can be guaranteed by Theorem 4.
**Algorithm 1:** MHDP-based mean estimation fusion.
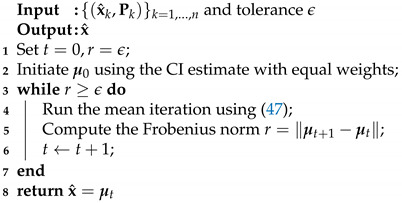

**Remark** **2.** *Similar to the GP method in [42], the fused scatter matrix estimate seems available to be set as*

(49) $$\begin{array}{c} P={\left(\sum_{k=1}^{n}\omega_{k}P_{k}^{-1}\right)}^{-1}, \end{array}$$

*since the fused mean estimate $\hat{x}$ has the CI form (45), and then the fused covariance estimate is naturally given as*

(50) $$\begin{array}{c} Q=\kappa_{h}P={\left(\sum_{k=1}^{n}\omega_{k}Q_{k}^{-1}\right)}^{-1} \end{array}$$

*with each local covariance $Q_{k}=\kappa_{h}P_{k}$. Nevertheless, (50) is reasonable to be replaced by the following two MHDP-based covariance fusion forms owing to its subjectively adopting the covariance fusion.*

### 4.2. Iterative Solution for Covariance Estimation Fusion

Define an auxiliary function

(51) $$\begin{array}{c} \psi_{k}\left(\Sigma \right)=\ell_{\Sigma }^{2}\left(p_{{\hat{\alpha }}_{k}},p\right), \end{array}$$

and then inserting the Rao distance (24) into (51) yields

(52) $$\begin{array}{c} \psi_{k}\left(\Sigma \right)=2b_{h}\mathrm{tr}\left(T_{k}^{2}\right)+\frac{4b_{h}-1}{4}{\mathrm{tr}}^{2}\left(T_{k}\right), \end{array}$$

where,

(53) $$\begin{array}{cc} {\overset{˘}{P}}_{k} & ={\hat{\alpha }}_{k}P_{k}, \end{array}$$

(54) $$\begin{array}{cc} T_{k} & =\mathrm{Log}\left({\overset{˘}{P}}_{k}^{-1}\Sigma \right). \end{array}$$

In the theorem given below, we derive the Riemannian gradient of $\psi_{k}\left(\Sigma \right)$ on $M_{\left[\hat{x},\cdot \right]}$.

**Theorem** **5.** *The Riemannian gradient of $\psi_{k}\left(\Sigma \right)$ on the submanifold $M_{\left[\hat{x},\cdot \right]}$ is given as*

(55) $$\begin{array}{c} \nabla \psi_{k}=2\Sigma T_{k}. \end{array}$$

**Proof.**  See Appendix E.    □

Furthermore, denoting the objective function in (41) as

(56) $$\begin{array}{c} \psi \left(\Sigma \right)=\sum_{k=1}^{n}\psi_{k}\left(\Sigma \right), \end{array}$$

and differentiating ψ(·) with respect to Σ, we have the Riemannian gradient vector

(57) $$\begin{array}{c} \nabla \psi =\sum_{k=1}^{n}\nabla \psi_{k}=2\Sigma \sum_{k=1}^{n}\mathrm{Log}\left({\overset{˘}{P}}_{k}^{-1}\Sigma \right). \end{array}$$

As a result, the minimizer P of (41) satisfies ∇ψ=0, i.e.,

(58) $$\begin{array}{c} \frac{1}{n}\sum_{k=1}^{n}\mathrm{Log}\left({\overset{˘}{P}}_{k}^{-1}\Sigma \right)=0. \end{array}$$

**Remark** **3.** *The average *Σ* of n symmetric positive-definite matrices ${\overset{˘}{P}}_{1},\ldots ,{\overset{˘}{P}}_{n}$, satisfying the barycentric Equation (58), is also called the Riemannian barycenter [59].*

In general, (58) cannot be solved explicitly due to the noncommutative nature on $S_{+}^{m}$, but we formulate the fixed point iteration for the scatter estimation fusion as follows:

(59) $$\begin{array}{c} \Sigma^{\left(t+1\right)}=\Sigma^{\left(t\right)}\cdot \mathrm{Exp}\left(\frac{1}{n}\sum_{k=1}^{n}\mathrm{Log}\left({\overset{˘}{P}}_{k}^{-1}\Sigma^{\left(t\right)}\right)\right), \end{array}$$

which is a very efficient iterative strategy for solving (58) with at least linear convergence (see [60] for a detailed proof). The fusion method above is called the MHDP-I method (i.e., Algorithm 2).
**Algorithm 2:** MHDP-I fusion algorithm.
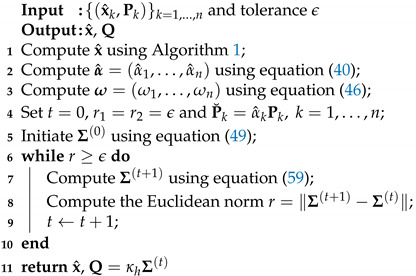


### 4.3. Explicit Solution for Covariance Estimation Fusion

The submanifold $M_{\left[\hat{x},\cdot \right]}$ with the fixed mean $\hat{x}$ can be identified with the symmetric positive-definite matrix space $S_{+}^{m}$ equipped with the same Riemannian metric induced by (23). Meanwhile, we can endow $S_{+}^{m}$ with a group structure depending on the following definition:

**Definition** **1.** *$S_{+}^{m}=\left(S_{+}^{m},⊙,i,e\right)$ is a commutative group whose group operations are defined as follows:*

*(i)*  *Multiplication operation: $g_{1}⊙g_{2}:=\mathrm{Exp}\left(\mathrm{Log}\left(g_{1}\right)+\mathrm{Log}\left(g_{2}\right)\right)$, for any $g_{1},g_{2}\in S_{+}^{m}$;*
*(ii)*  *Inverse operation: $i\left(g\right):=g^{-1}$, for any $g\in S_{+}^{m}$;*
*(iii)*  *Identity element: $e=I_{m}$.*

It is easy to verify that the two operations ⊙ and *i* are compatible with the Riemannian structure on $S_{+}^{m}$ (see, e.g., [61]), hence $S_{+}^{m}$ is a Lie group.

As is well known, the Lie group is locally equivalent to a linear space, in which the local neighborhood of any element can be described by its tangent space. The tangent space $T_{e}M_{\left[\hat{x},\cdot \right]}$ at the identity element $e=I_{m}$, also called Lie algebra g, is indeed isomorphic to the space $S^{m}$ of real symmetric matrices. Theoretically, the retraction mapping R(·) establishes a vector space isomorphism from $\left(S^{m},+\right)$ to $\left(M_{\left[\hat{x},\cdot \right]},⊙\right)$. Then, we endow $M_{\left[\hat{x},\cdot \right]}$, namely $S_{+}^{m}$, with a “Lie vector space structure”.

It is important to stress that, based on the linear structure of Lie algebra, we can handle the covariance/scatter estimation fusion with the Euclidean operation, i.e., the arithmetic averaging in the Log-Euclidean domain, instead of the Riemannian one. It is explained as follows:

(i) Move the MHDPs ${\overset{˘}{P}}_{1},\ldots ,{\overset{˘}{P}}_{n}$ into some neighborhood of the identity element $e=I_{m}\in M_{\left[\hat{x},\cdot \right]}$ via using a left translation $L_{P}\left(\cdot \right)$ by the sought-for scatter estimate P to obtain $L_{P}\left({\overset{˘}{P}}_{k}\right)=P^{-1}⊙{\overset{˘}{P}}_{k}$. Then, all $L_{P}\left({\overset{˘}{P}}_{k}\right)$ can be shifted to g by the lifting mapping $R_{e}^{-1}\left(\cdot \right)$ at *e*, obtaining the vectors $R_{e}^{-1}\left(L_{P}\left({\overset{˘}{P}}_{k}\right)\right)$ for k=1,…,n.
(ii) The arithmetic average of $R_{e}^{-1}\left(L_{P}\left({\overset{˘}{P}}_{k}\right)\right)$ is the best way to minimize the total displacements from each $P_{k}$ to the sought-for P on the Lie algebra g owing to the linear structure of g (see, e.g., [60]), hence $\frac{1}{n}\sum_{k=1}^{n}R_{e}^{-1}\left(P^{-1}⊙{\overset{˘}{P}}_{k}\right)=0,$ or equivalently,
  (60) $$\begin{array}{c} P=P⊙R_{e}\left(\frac{1}{n}\sum_{k=1}^{n}R_{e}^{-1}\left(P^{-1}⊙{\overset{˘}{P}}_{k}\right)\right). \end{array}$$

Let the retraction mapping $R_{e}\left(\cdot \right)=\exp_{e}\left(\cdot \right)$ and the lifting mapping $R_{e}^{-1}\left(\cdot \right)=\log_{e}\left(\cdot \right)$ on $M_{\left[\hat{x},\cdot \right]}$, whose analytical expressions are given in the theorem below.

**Theorem** **6.** *The following properties hold:*

*(i)*  *Let H be a non-singular matrix. The metric on $M_{\left[\hat{x},\cdot \right]}$ originated from (23) is invariant under the congruent transformation: $\Sigma \to H^{T}\Sigma H$ for any $\Sigma \in S_{+}^{m}$.*
*(ii)*  *The exponential mapping and logarithmic mapping on $M_{\left[\hat{x},\cdot \right]}$ are, respectively, given by*
(61) $$\begin{array}{c} \exp_{\Sigma_{1}}\left(\nu \right)=\Sigma_{1}\mathrm{Exp}\left(\Sigma_{1}^{-1}\nu \right), \end{array}$$
(62) $$\begin{array}{c} \log_{\Sigma_{1}}\left(\Sigma_{2}\right)=\Sigma_{1}\mathrm{Log}\left(\Sigma_{1}^{-1}\Sigma_{2}\right), \end{array}$$
  *for any $\Sigma_{1},\Sigma_{2}\in M_{\left[\hat{x},\cdot \right]}$ and $\nu \in T_{\Sigma_{1}}M_{\left[\hat{x},\cdot \right]}$.*

**Proof.**  See Appendix F.    □

Substituting (61) and (62) into (60) yields

(63) $$\begin{array}{cc} P & =P⊙\mathrm{Exp}\left(\frac{1}{n}\sum_{k=1}^{n}\mathrm{Log}\left(P^{-1}⊙{\overset{˘}{P}}_{k}\right)\right). \end{array}$$

Then, by $P^{-1}⊙{\overset{˘}{P}}_{k}=\mathrm{Exp}\left(\mathrm{Log}{\overset{˘}{P}}_{k}-\mathrm{Log}P\right)$ and the definition of ⊙, we have the fused scatter matrix estimate

(64) $$\begin{array}{c} P=\mathrm{Exp}\left(\frac{1}{n}\sum_{k=1}^{n}\mathrm{Log}\left({\overset{˘}{P}}_{k}\right)\right), \end{array}$$

and the fused covariance matrix estimate

(65) $$\begin{array}{c} Q=\kappa_{h}\mathrm{Exp}\left(\frac{1}{n}\sum_{k=1}^{n}\mathrm{Log}\left({\overset{˘}{P}}_{k}\right)\right). \end{array}$$

**Remark** **4.** *As shown in [60], the explicit scatter matrix estimate (64) coincides with the arithmetic mean in the log-domain, i.e., the Log-Euclidean Fréchet mean.*

**Figure 2:**
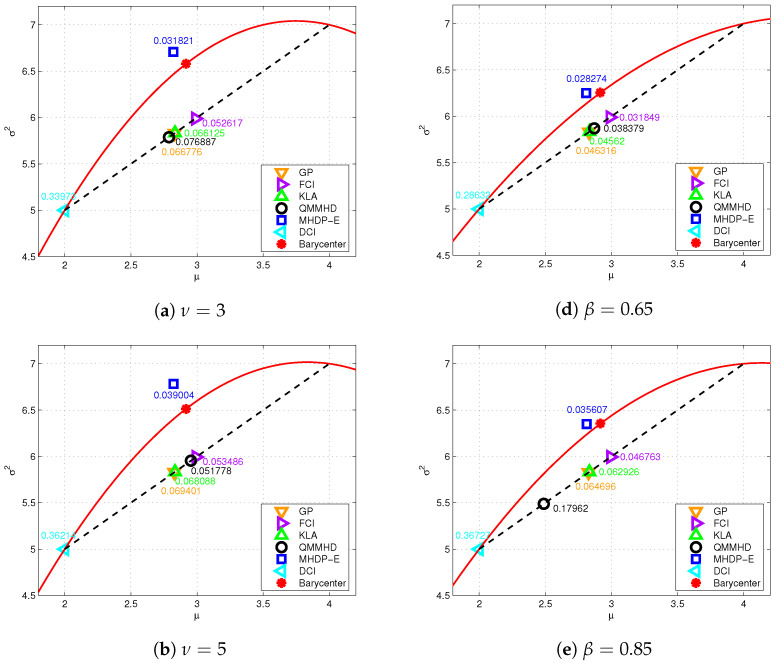

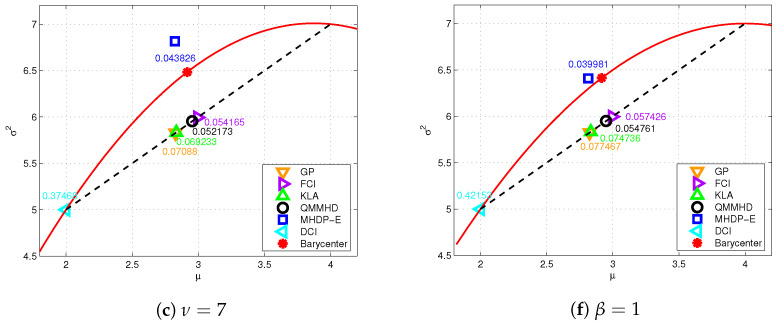
Six subfigures are depicted for the geodesic distance between the fused density and the informative barycenter. The various fused results are labeled by the specified marks. Subfigures (**a**–**c**) correspond to MTDs with DOF parameters ν=3,5 and 7, respectively. Subfigures (**d**–**f**) correspond to MGGDs with shape parameters β=0.65,0.85 and 1, respectively. In each subfigure, the geodesic distances between the informative barycenter and the fused densities are displayed, and the geodesic between the two local densities is represented by the solid red curve.

In summary, the fusion method above is called the MHDP-E method (i.e., Algorithm 3). For comparison, the MHDP-I and MHDP-E fusion algorithms have the same mean fusion (see Algorithm 1), but the latter possesses an explicit form for the fused covariance matrix estimate (65). In addition, many existing fusion methods have identical forms as the CI, but differ from each other in the weights. For example, the KLA has equal weights 1/N, while many others, including DCI, FCI, GP and QMMHD, have their own weights, depending on local estimates. Taking two sensors tracking a one-dimensional target for instance, the fused estimates for the KLA, DCI, FCI, GP and QMMHD theoretically lie on the (straight) Euclidean line segment between two local estimates from sensors, as shown in Figure 2 of Section 5.1. However, the MED manifold M with the Riemannian structure is generally not flat [47], so the proposed MHDP-I and MHDP-E are more reasonable owing to their improving covariance estimation fusion based on this geometric structure.
**Algorithm 3:** MHDP-E fusion algorithm. **Input:** ${\left\{\left({\hat{x}}_{k},P_{k}\right)\right\}}_{k=1,\ldots ,n}$ **Output**: $\hat{x}$, Q **1** Compute $\hat{x}$ using Algorithm 1; **2** Compute $\hat{\alpha }=\left({\hat{\alpha }}_{1},\ldots ,{\hat{\alpha }}_{n}\right)$ using Equation (40); **3** Set ${\overset{˘}{P}}_{k}={\hat{\alpha }}_{k}P_{k},\;k=1,\ldots ,n$; **4** Compute Q using Equation (65); **5**
**return** $\hat{x}$, Q

## 5. Numerical Examples

In this section, we provide two numerical examples to show the performance of two fusion methods MHDP-I and MHDP-E, including static and dynamic target tracking. The traditional fusion algorithms DCI [16], FCI [15], and KLA [18], and two information-geometric methods GP [42] and QMMHD [43] are taken for comparison.

### 5.1. Static Target Tracking

To intuitively evaluate the performance of these distributed fusion methods, we consider two local estimates $\left({\hat{x}}_{1},P_{1}\right)=\left(2,5\right)$ and $\left({\hat{x}}_{2},P_{2}\right)=\left(4,7\right)$ of a static target from two local sensors, similar to the settings in [42]. Note that the MHDP- I and MHDP-E methods have the same covariance fusion due to the commutability of two first-order matrices. In Figure 2, six subfigures are drawn to display the geodesic distance between the fused density and the informative barycenter under six different MED assumptions, respectively. Here, the informative barycenter as the optimal solution of (29) is an arbitrary point on the geodesic segment linking $\left({\hat{x}}_{1},P_{1}\right)$ and $\left({\hat{x}}_{2},P_{2}\right)$, and we uniquely determine it by minimizing the sum of its squared distances to the two local estimates. Since the MHDP-I and MHDP-E are developed based on the relaxation of (29), learning about the informative barycenter can improve our understanding of their fusion performance as the heavy-tailed level varies.

(i) The MTD has the degree of freedom (DOF) parameter ν>2 (the higher ν, the lower the heavy-tailed level), and is almost equivalent to the Gaussian distribution as ν tends to infinity. As depicted in subfigures (a), (b) and (c), the MHDP-E is consistently superior to other methods regardless of the DOF, since the fused density of the MHDP-E is closest to the informative barycenter.
(ii) The MGGD has the shape parameter β>0 (the higher β, the lower the heavy-tailed level) and corresponds to the Gaussian distribution when β=1. Similar to the MTD, subfigures (d), (e) and (f) show the MHDP-E outperforms other fusion methods for different shape parameters.

Moreover, the aforementioned fusion methods DCI, FCI, KLA, GP and QMMHD have the same form as (45), but with different weights in general. Thus in Figure 2 their fused estimates lie on the (straight) Euclidean segment between $\left({\hat{x}}_{1},P_{1}\right)$ and $\left({\hat{x}}_{2},P_{2}\right)$, while the MHDP-E puts its estimate fairly close to the (curved) geodesic segment. Therefore, the MHDP-I and MHDP-E are considered more reasonable because the MED manifold M is indeed non-Euclidean.

### 5.2. Dynamic Target Tracking

Consider a distributed dynamic system with *n* sensors and one fusion center for tracking a two-dimensional moving target:

(66) $$\begin{array}{cc} x_{k+1} & =\left[\begin{array}{cc} 1 & T\\ 0 & 1 \end{array}\right]x_{k}+\left[\begin{array}{c} T^{2}/2\\ T \end{array}\right]w_{k}, \end{array}$$

(67) $$\begin{array}{cc} z_{k}^{i} & =h_{k}^{i}x_{k}+v_{k}^{i},\;i=1,\ldots ,n_{0}, \end{array}$$

(68) $$\begin{array}{cc} z_{k}^{i} & =H_{k}^{i}x_{k}+v_{k}^{i},\;i=n_{0}+1,\ldots ,n, \end{array}$$

where there are $n_{0}$ sensors for one-dimensional measurements and $n-n_{0}$ sensors for multi-dimensional measurements, the state vector $x_{k}$ consists of the position and velocity, and the sampling interval T=1s. As [49,50] for more practical scenarios, the outlier contaminated state and measurement noises are modeled as

(69) $$\begin{array}{cc} w_{k} & ∼\left\{\begin{array}{cc} N(0,Q), & w.p.\;0.9,\\ N(0,200Q), & w.p.\;0.1, \end{array}\right. \end{array}$$

(70) $$\begin{array}{cc} v_{k}^{i} & ∼\left\{\begin{array}{cc} N(0,R_{k}^{i}), & w.p.\;0.9,\\ N(0,uR_{k}^{i}), & w.p.\;0.1, \end{array}\right.\;i=1,\ldots ,n_{0}, \end{array}$$

(71) $$\begin{array}{cc} v_{k}^{i} & ∼\left\{\begin{array}{cc} N(0,R_{k}^{i}), & w.p.\;0.9,\\ N(0,uR_{k}^{i}), & w.p.\;0.1, \end{array}\right.\;i=n_{0}+1,\ldots ,n, \end{array}$$

where “w.p.” denotes “with probability”.

The local state estimate ${\hat{x}}_{i}\left(k|k\right)$ and scatter matrix $P_{i}\left(k|k\right)$ of estimation error are computed by using the specified filter, when the *i*-th sensor obtains its own measurement at the instant *k*. The fusion center fuses all local estimates from the sensors to obtain the final estimate $\left(\hat{x}\left(k|k\right),P\left(k|k\right)\right)$ by applying some specified fusion methods, and then transmits it back to each local sensor. To demonstrate the performance of various fusion methods, i.e., the DCI, FCI, KLA, GP, QMMHD, MHDP-I and MHDP-E, we use the Student’s *t* based filter (STF) [49] and the Gaussian–Student’s *t* mixture distribution based Kalman filter (GSTMDKF) [41] to compare the root mean squared errors (RMSEs) of the fused results in the following two cases:

(i) Case I (two sensors, i.e., $n_{0}=n=2$): The variances Q=1 and $R_{k}^{1}=R_{k}^{2}=16$, the measurement matrices
  (72) $$\begin{array}{c} h_{k}^{1}=\left[1,0\right],\;h_{k}^{2}=\left[0,1\right], \end{array}$$
  and the two filters are initialized as
  (73) $$\begin{array}{c} \hat{x}\left(0|0\right)=\left[\begin{array}{c} 0\\ 5 \end{array}\right],\;P\left(0|0\right)=\left[\begin{array}{cc} 10 & \\ \; & 25 \end{array}\right]. \end{array}$$
(ii) Case II (three sensors, i.e., $n_{0}=2,\;n=3$): The system parameters $Q,R_{k}^{1},R_{k}^{2},h_{k}^{1},h_{k}^{2},\hat{x}\left(0|0\right),P\left(0|0\right)$ have the same settings as Case I, and also
  (74) $$\begin{array}{c} H_{k}^{3}=\left[\begin{array}{cc} 1 & \\ \; & 1 \end{array}\right],\;R_{k}^{3}=\left[\begin{array}{cc} 16 & 4\\ 4 & 6 \end{array}\right]. \end{array}$$

It is seen from (69)–(71) that the process and observation noises follow heavy-tailed distributions. The STF models the initial state vector and the noise distributions with u=200 as follows:

(75) $$\begin{array}{cc} x_{0} & ∼\mathrm{MTD}(\hat{x}\left(0|0\right),P\left(0|0\right),\nu_{1}), \end{array}$$

(76) $$\begin{array}{cc} w_{k} & ∼\mathrm{MTD}(0,Q,\nu_{2}), \end{array}$$

(77) $$\begin{array}{cc} v_{k}^{i} & ∼\mathrm{MTD}\left(0,R_{k}^{i},\nu_{3}\right),\;i=1,\ldots ,n_{0}, \end{array}$$

(78) $$\begin{array}{cc} v_{k}^{i} & ∼\mathrm{MTD}\left(0,R_{k}^{i},\nu_{4}\right),\;i=n_{0}+1,\ldots ,n. \end{array}$$

By (75)–(78), each local posterior density can be represented by the MTD with the DOF parameter ν=3, i.e., $\nu_{i}=3,\;i=1,\ldots ,4$. In the GSTMDKF setup, a Gaussian–Student’s *t* mixture distribution (see [41] for more details) with the fixed DOF 3 is used to model the heavy-tailed noises (69)–(71) with u=100, the initial state vector follows a Gaussian distribution, i.e., $x_{0}∼N\left(\hat{x}\left(0|0\right),P\left(0|0\right)\right)$, and other parameters are the same as those in [41]. As explained in [41], the GSTM distribution has the same efficiency as the Student’s *t* distribution in the presence of outliers, and thus our proposed MHDP-E and MHDP-I methods fuse all posterior densities by modeling them as the MTDs with the common DOF parameter 3. Note that the DCI, FCI, KLA, and GP only utilize the first two moments (i.e., $\hat{x}\left(k|k\right)$ and κP(k|k) for the *i*-th local posterior density), and the FCI, KLA and GP fusion methods are developed under Gaussian assumption so that the posterior density for the *i*-th sensor can be modeled as $N({\hat{x}}_{i}\left(k|k\right),\kappa P_{i}\left(k|k\right))$.

**Figure 3:**
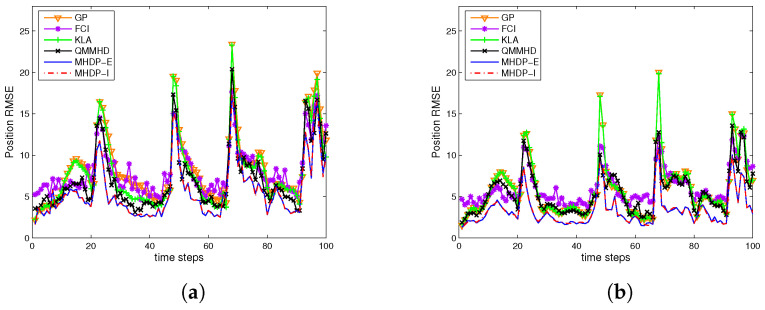

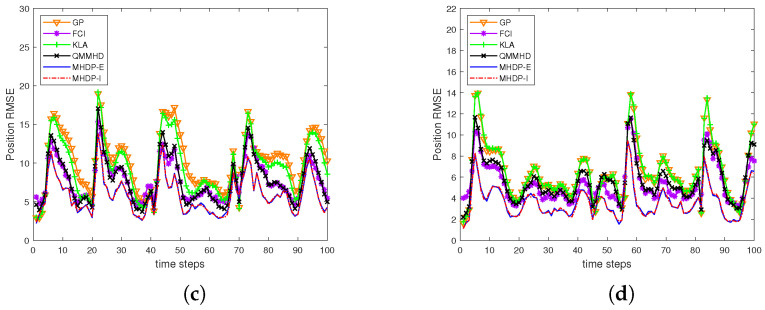
The position RMSEs of fused results vs. the instant *k* from 1 to 100 by (**a**) the STF in Case I; (**b**) the STF in Case II; (**c**) the GSTMDKF in Case I; and (**d**) the GSTMDKF in Case II.

By adopting two different filters STF and GSTMDKF, Figure 3 shows the RMSEs of the position over 100 time steps and 500 Monte Carlo runs in two different cases using the aforementioned fusion methods, while the DCI does not appear in Figure 3 owing to it behaving worst. We can see that the proposed MHDP-I and MHDP-E are consistently better than other fusion methods when different filtering methods are adopted. Moreover, Table 1 reports the averaged root mean squared errors (ARMSEs) of the various methods in both cases. It is evident that the performance of all fusion methods in three-sensor example has a significant improvement over that in the two-sensor case, and also the MHDP-I performs slightly better than the MHDP-E when using the GSTMDKF. However in contrast to the MHDP-I, the MHDP-E has lower computational complexity due to the explicit expression for the fused covariance estimate.

## 6. Conclusions

In this work, we formulate an information-geometric fusion criterion, taking the geodesic distance as the loss function, and implement the fusion of local posterior densities on the MED manifold endowed with the Fisher metric. Two distributed estimation fusion algorithms MHDP-I and MHDP-E are proposed by using the minimal Manhattan distance instead of the geodesic distance on the manifold of MEDs, which both have the same mean estimation fusion, but differ in the form of covariance estimation fusion. On the MED submanifold with a fixed mean, the MHDP-I fuses all MHD-based projections of local posterior densities by minimizing the squared geodesic distances from a sought-for fused density. We have developed a robust fixed point iterative algorithm with theoretical convergence to compute the covariance estimate of such fused density. By introducing a specified Lie algebraic structure on the aforementioned submanifold and deriving its exponential and logarithmic mappings, the MHDP-E has provided an explicit expression for the fused covariance estimate. Numerical examples have shown better performance of the two proposed information-geometric methods than five other fusion methods.

## Figures and Tables

**Table 1:** The Position ARMSEs of seven fusion methods in both cases by the STF and the GSTMDKF.

Method	STF		GSTMDKF	
	Case I	Case II	Case I	Case II
DCI	28.99	26.76	46.01	35.84
GP	8.54	5.93	10.35	6.47
FCI	8.23	6.15	7.82	5.36
KLA	7.92	5.96	9.47	6.49
QMMHD	7.06	5.45	7.78	5.65
MHDP-E	5.72	3.35	5.91	3.64
MHDP-I	5.72	3.36	5.90	3.63

## Appendix A. Proof of Theorem 1

Let $c_{h}=8a_{h}/\left(12b_{h}-1\right)$ and the variable transformation

(A1) $$\begin{array}{c} \overset{˘}{\mu }=\sqrt{c_{h}}\mu . \end{array}$$

By using (13), the geodesic equations on the MED manifold with the coordinate system (μ,Σ) can be rewritten as

(A2) $$\begin{array}{cc} \ddot{\overset{˘}{\mu }}-\dot{\Sigma }\Sigma^{-1}\dot{\overset{˘}{\mu }} & =0, \end{array}$$

(A3) $$\begin{array}{cc} \ddot{\Sigma }+{\dot{\overset{˘}{\mu }}}^{2}-\Sigma^{-1}{\dot{\Sigma }}^{2} & =0. \end{array}$$

Then, the squared line element along the geodesic becomes

(A4) $$\begin{array}{cc} d{\overset{˘}{s}}^{2} & ={\left(d\overset{˘}{\mu }\right)}^{2}\Sigma^{-1}+\frac{1}{2}{\left(\Sigma^{-1}d\Sigma \right)}^{2}\\ \; & =\frac{2}{12b_{h}-1}\left(4a_{h}{\left(d\mu \right)}^{2}\Sigma^{-1}+\left(3b_{h}-1/4\right){\left(\Sigma^{-1}d\Sigma \right)}^{2}\right). \end{array}$$

Compared with (11), we obtain $\overset{˘}{s}=\sqrt{2/(12b_{h}-1)}s$, where *s* is the arc length parameter before the transformation (A1). Therefore, by Theorem 6.5 in [62], the geodesic distance between $p_{1}$ and $p_{2}$ is given as

(A5) $$\begin{array}{c} \ell_{F}=d_{h}\cdot \mathrm{arccosh}\left(\frac{c_{h}{\left(\mu_{2}-\mu_{1}\right)}^{2}+2\left(\sigma_{1}^{2}+\sigma_{2}^{2}\right)}{4\sigma_{1}\sigma_{2}}\right) \end{array}$$

with $d_{h}=\sqrt{12b_{h}-1}$. To that end, using Theorem 6.2 in [62], we obtain the geodesic curve on the MED manifold as

(A6) $$\begin{array}{cc} \mu (s) & =\left(\delta_{1}+2\delta_{2}\tanh\left(s/d_{h}+\varepsilon \right)\right)/\sqrt{c_{h}}, \end{array}$$

(A7) $$\begin{array}{cc} \sigma^{2}\left(s\right) & =2\delta_{2}^{2}\cosh^{-2}\left(s/d_{h}+\varepsilon \right), \end{array}$$

where $\delta_{1},\delta_{2}$ and ε can be calculated by boundary conditions. Note that for the case of $\mu_{1}=\mu_{2}$, the geodesic given by (15) and (16) are easily obtained by Equation (6.4) in [62].

## Appendix B. Proof of Theorem 2

Define an auxiliary function

(A8) $$\begin{array}{c} f\left(\alpha \right)=\frac{{\left(cd_{M}+2\right)}^{2}}{8\alpha }+\frac{\alpha }{2}+\frac{cd_{M}}{2},\;\alpha \in R_{+}. \end{array}$$

It is easy to see from (26) and (A8) that the minimization for (31) is equivalent to minimizing f(α) with respect to $\alpha \in R_{+}$. We have thus $\ell_{M}\left(p_{0},M_{\left[\mu ,\cdot \right]}\right)=\min_{\alpha \in R_{+}}\ell_{\mu }\left(p_{0},p_{\alpha }\right)$, the optimal scale factor

(A9) $$\begin{array}{cc} {\hat{\alpha }}_{0} & :=\underset{\alpha \in R_{+}}{\arg\min}\ell_{\mu }\left(p_{0},p_{\alpha }\right)\\ \; & =\underset{\alpha \in R_{+}}{\arg\min}\left(\frac{{\left(cd_{0}+2\right)}^{2}}{8\alpha }+\frac{\alpha }{2}+\frac{cd_{0}}{2}\right)\\ \; & =\frac{cd_{0}}{2}+1 \end{array}$$

with $d_{0}={\left({\hat{x}}_{0}-\mu \right)}^{T}P_{0}^{-1}\left({\hat{x}}_{0}-\mu \right)$ and the unique MHDP $p_{{\hat{\alpha }}_{0}}={\mathrm{EL}}_{h}^{m}\left(\mu ,{\hat{\alpha }}_{0}P_{0}\right)$. Therefore, substituting (A9) into (26) yields the MHDPD (32).

## Appendix C. Proof of Theorem 3

By the Lagrange multiplier method, the minimizer $\hat{x}$ of (44) must satisfy the following equality:

(A10) $$\begin{array}{c} \sum_{k=1}^{n}\dot{\phi }\left({\overset{˘}{d}}_{k}\left(\mu \right)\right)P_{k}^{-1}\left(\mu -{\hat{x}}_{k}\right)=0. \end{array}$$

Taking further derivation, we immediately have (45) and (46). Additionally, it is obvious that $\dot{\phi }\left(x\right)\geq 0$ for x≥1, and thus each $\omega_{k}\in \left[0,1\right]$.

## Appendix D. Proof of Theorem 4

Define an auxiliary function

(A11) $$\begin{array}{c} \varphi \left(x\right)=\frac{x_{1}+x}{\sqrt{x_{1}^{2}-1}+\sqrt{x^{2}-1}},\;x\in \left(1,+\infty \right) \end{array}$$

with $x_{1}>1$. It is easy to know $\dot{\varphi }\left(x\right)\leq 0$, and thus we have the inequality

(A12) $$\begin{array}{c} \frac{x_{1}+x_{2}}{\sqrt{x_{1}^{2}-1}+\sqrt{x_{2}^{2}-1}}\leq \frac{x_{1}}{\sqrt{x_{1}^{2}-1}} \end{array}$$

for $x_{2}\geq x_{1}>1$.

Furthermore, define two functions of $\mu \in R^{m}$ as follows:

(A13) $$\begin{array}{cc} \overset{˘}{\phi }\left(\mu \right) & =\sum_{k=1}^{n}\phi \left({\overset{˘}{d}}_{k}\left(\mu \right)\right)=\sum_{k=1}^{n}\mathrm{arccosh}\left({\overset{˘}{d}}_{k}\left(\mu \right)\right), \end{array}$$

(A14) $$\begin{array}{cc} {\overset{˘}{\phi }}_{\mu_{t}}\left(\mu \right) & =\overset{˘}{\phi }\left(\mu_{t}\right)+\sum_{k=1}^{n}\frac{{\overset{˘}{d}}_{k}\left(\mu \right)-{\overset{˘}{d}}_{k}\left(\mu_{t}\right)}{\sqrt{{\overset{˘}{d}}_{k}^{2}\left(\mu_{t}\right)-1}}. \end{array}$$

Utilizing two inequalities log(x)≤x−1 and (A12), we obtain

(A15) $$\begin{array}{cc} \; & \mathrm{arccosh}\left({\overset{˘}{d}}_{k}\left(\mu \right)\right)-\mathrm{arccosh}\left({\overset{˘}{d}}_{k}\left(\mu_{t}\right)\right)\\ \; & \leq \frac{{\overset{˘}{d}}_{k}\left(\mu \right)+\sqrt{{\overset{˘}{d}}_{k}^{2}\left(\mu \right)-1}}{{\overset{˘}{d}}_{k}\left(\mu_{t}\right)+\sqrt{{\overset{˘}{d}}_{k}^{2}\left(\mu_{t}\right)-1}}-1\\ \; & =\left({\overset{˘}{d}}_{k}\left(\mu \right)-{\overset{˘}{d}}_{k}\left(\mu_{t}\right)\right)\frac{1+\frac{{\overset{˘}{d}}_{k}\left(\mu \right)+{\overset{˘}{d}}_{k}\left(\mu_{t}\right)}{\sqrt{{\overset{˘}{d}}_{k}^{2}\left(\mu \right)-1}+\sqrt{{\overset{˘}{d}}_{k}^{2}\left(\mu_{t}\right)-1}}}{{\overset{˘}{d}}_{k}\left(\mu_{t}\right)+\sqrt{{\overset{˘}{d}}_{k}^{2}\left(\mu_{t}\right)-1}}\\ \; & \leq \frac{{\overset{˘}{d}}_{k}\left(\mu \right)-{\overset{˘}{d}}_{k}\left(\mu_{t}\right)}{\sqrt{{\overset{˘}{d}}_{k}^{2}\left(\mu_{t}\right)-1}}. \end{array}$$

By (A13), (A14) and (A15), it follows that $\overset{˘}{\phi }\left(\mu \right)\leq {\overset{˘}{\phi }}_{\mu_{t}}\left(\mu \right)$ and $\overset{˘}{\phi }\left(\mu_{t}\right)={\overset{˘}{\phi }}_{\mu_{t}}\left(\mu_{t}\right)$, and thus $\overset{˘}{\phi }\left(\mu \right)$ is majorized by ${\overset{˘}{\phi }}_{\mu_{t}}\left(\mu \right)$ at point $\mu_{t}$ under the majorization–minimization framework [63].

Consequently, setting the gradient of ${\overset{˘}{\phi }}_{\mu_{t}}\left(\mu \right)$ with respect to μ as zero, we conclude that

(A16) $$\begin{array}{c} \sum_{k=1}^{n}\frac{P_{k}^{-1}\left(\mu -{\hat{x}}_{k}\right)}{\sqrt{{\overset{˘}{d}}_{k}^{2}\left(\mu_{t}\right)-1}}=0, \end{array}$$

and then the fixed point iteration (47) for the mean estimate $\hat{x}$ follows from (A16).

## Appendix E. Proof of Theorem 5

**Lemma** **A1** (See [52]). *If X(t) is an invertible real matrix-valued function of real variable t and its eigenvalues lie on the positive real line, then*

(A17) $$\begin{array}{c} \frac{d}{dt}\mathrm{tr}\left({\mathrm{Log}}^{2}X\left(t\right)\right)=2\mathrm{tr}\left(\mathrm{Log}X\left(t\right)\;X^{-1}\left(t\right)\frac{d}{dt}X\left(t\right)\right). \end{array}$$

Let γ(t;θ,ν) denote the geodesic emanating from the point θ=Σ at t=0 in an arbitrary direction $\nu \in T_{\theta }M_{\left[\hat{x},\cdot \right]}$. Inspired by the method in the proof of Lemma A1 in [52], we can easily know

(A18) $$\begin{array}{cc} \frac{d}{dt}\mathrm{tr}\left(\mathrm{Log}\gamma (t)\right) & =\mathrm{tr}\left(\int_{0}^{1}{\left(\left(\gamma \left(t\right)-I\right)s+I\right)}^{-2}ds\cdot \frac{d\gamma (t)}{dt}\right)\\ \; & =\mathrm{tr}\left(\gamma^{-1}\left(t\right)\frac{d}{dt}\gamma \left(t\right)\right). \end{array}$$

Applying (A17) and (A18), and differentiating $\psi_{k}\left(\gamma \left(t\right)\right)$ with respect to *t*, we have

(A19) $$\begin{array}{cc} \frac{d}{dt}\psi_{k}\left(\gamma \left(t\right)\right)= & 4b_{h}\mathrm{tr}\left(T_{k}\left(t\right)\gamma^{-1}\left(t\right)\frac{d\gamma (t)}{dt}\right)\\ \; & +\left(2b_{h}-\frac{1}{2}\right)\mathrm{tr}\left(T_{k}\left(t\right)\right)\mathrm{tr}\left(\gamma^{-1}\left(t\right)\frac{d\gamma (t)}{dt}\right), \end{array}$$

where $T_{k}\left(t\right):=\mathrm{Log}\left({\overset{˘}{P}}_{k}^{-1}\gamma \left(t\right)\right)$. As a result,

(A20) $$\begin{array}{cc} \frac{d}{dt}\psi_{k}\left(\gamma \left(t\right)\right){|}_{t=0}= & 2b_{h}\mathrm{tr}\left(2T_{k}\Sigma^{-1}\nu \right)+\frac{4b_{h}-1}{4}\mathrm{tr}\left(2T_{k}\right)\mathrm{tr}\left(\Sigma^{-1}\nu \right). \end{array}$$

According to the arbitrariness of vector ν and the Riemannian inner product (23), we obtain the Riemannian gradient

(A21) $$\begin{array}{c} ▽\psi_{k}=2\Sigma T_{k}. \end{array}$$

## Appendix F. Proof of Theorem 6

(i) From the inner product (23), we have the squared line element

(A22) $$\begin{array}{c} ds^{2}=2b_{h}\mathrm{tr}{\left(\Sigma^{-1}d\Sigma \right)}^{2}+\frac{4b_{h}-1}{4}{\mathrm{tr}}^{2}\left(\Sigma^{-1}d\Sigma \right). \end{array}$$

By taking the congruent transformation $\Sigma \to H^{T}\Sigma H$, it is not difficult to see that the squared infinitesimal distance (A22) is invariant.

(ii) Suppose

(A23) $$\begin{array}{c} r_{k}=\log\lambda_{k},\;k=1,\ldots ,m, \end{array}$$

where $\lambda_{k}$ are the eigenvalues of $\Sigma_{1}^{-1}\Sigma_{2}$. There exists a nonsingular matrix H such that

(A24) $$\begin{array}{c} H^{T}\Sigma_{1}H=I_{m},H^{T}\Sigma_{2}H=\Lambda , \end{array}$$

where $\Lambda =\mathrm{diag}(\left[\lambda_{1},\ldots ,\lambda_{m}\right])$. According to the geodesic from $I_{m}$ to Λ on $M_{\left[\hat{x},\cdot \right]}$

(A25) $$\begin{array}{c} r_{k}\left(t\right)=a_{k}t+b_{k},\;k=1,\ldots ,m \end{array}$$

with initial conditions $r_{k}\left(0\right)=0$ and $r_{k}\left(1\right)=r_{k}$ (see [48]), we have $a_{k}=\log\lambda_{k}$, $b_{k}=0$ and then the geodesic direction

(A26) $$\begin{array}{cc} \dot{\Lambda }\left(0\right) & =\mathrm{Log}(\Lambda ). \end{array}$$

Let γ(t),t∈[0,1] be the shortest geodesic curve connecting two points $\Sigma_{1}$ and $\Sigma_{2}$ on $M_{\left[\hat{x},\cdot \right]}$. Due to the invariance of geodesic distance under the congruent transformation, we have

(A27) $$\begin{array}{c} \dot{\Lambda }\left(0\right)=H^{T}\dot{\gamma }\left(0\right)H, \end{array}$$

and thus substituting (A26) into (A27) yields

(A28) $$\begin{array}{cc} \dot{\gamma }\left(0\right) & ={\left(H^{T}\right)}^{-1}\mathrm{Log}\left(\Lambda \right)H^{-1}. \end{array}$$

Moreover, by the first equality in (A24) it follows that $\Sigma_{1}={\left(H^{T}\right)}^{-1}H^{-1}$, and inserting it into (A28), we obtain

(A29) $$\begin{array}{cc} \dot{\gamma }\left(0\right) & ={\left(H^{T}\right)}^{-1}\cdot \mathrm{Log}\left(H^{T}\Sigma_{2}H\right)\cdot H^{-1}\\ \; & ={\left(H^{T}\right)}^{-1}\cdot \mathrm{Log}\left(H^{-1}\Sigma_{1}^{-1}\Sigma_{2}H\right)\cdot H^{-1}\\ \; & ={\left(HH^{T}\right)}^{-1}\cdot \mathrm{Log}\left(\Sigma_{1}^{-1}\Sigma_{2}\right)\\ \; & =\Sigma_{1}\cdot \mathrm{Log}\left(\Sigma_{1}^{-1}\Sigma_{2}\right), \end{array}$$

i.e., the logarithmic mapping

(A30) $$\begin{array}{c} \log_{\Sigma_{1}}\left(\Sigma_{2}\right)=\Sigma_{1}\mathrm{Log}\left(\Sigma_{1}^{-1}\Sigma_{2}\right). \end{array}$$

As a result, replacing the left hand side of (A30) with $\nu \in T_{\Sigma_{1}}M_{\left[\hat{x},\cdot \right]}$, we obtain the exponential mapping

(A31) $$\begin{array}{c} \exp_{\Sigma_{1}}\left(\nu \right)=\Sigma_{1}\mathrm{Exp}\left(\Sigma_{1}^{-1}\nu \right). \end{array}$$
